# Aluminium

**Published:** 1857-06

**Authors:** A. Snowden Piggot


### Selections.

### ALUMINIUM.

BY A. SNOWDEN PIGGOT, M. D

For some time past this metallic basis of the common earth contained in all our clays, has been attracting the attention of chemists, especially in France. At last, results have been attained which have induced M. Dumas to state to the Academy, that the manufacture of the article has reached a point at which it may be taken out of the hands of science and handed over to artizans. He even states, that the manufacture is actually carried on by workmen in a small shop, in the Faubourg St. Jacques, at Paris, connected with a manufacture of chemical products. Having reached this point, we may expect soon to see it in commerce, and as its lightness and unalterability under common influences, appear to recommend it to dentists, we think we shall be doing a service to our readers, by putting them in possession of the principal facts connected with its manufacture, its chemical properties and its alloys.

On the 6th of February, 1854, Sainte-Claire Deville published in the *Comptes Rendus*, a paper in which he called attention to the readiness with which this metal might be obtained. He recalled the fact, that the eminent German technological chemist, Wohler, had obtained the metal in the form of a powder, by treating its chloride with potassium, the metallic basis of potash. He stated, that by modifying the process a little, and using sodium instead of potassium, it was easy to obtain aluminium in globules. He suggested the great advantages which must accrue to the arts from the introduction into commerce of a metal as white and unchangeable as silver, which does not tarnish in the air, which possesses the desirable qualities of easy fusibility, malleability and toughness. Its lightness being less than that of glass, and its inexhaustible abundance still further recommended it to his attention.

The Academy were so impressed with the results of this laboratory experiment, that they gave the patient investigator facilities for more extensive researches, which were still, however, conducted on a small scale. He used large glass tubes, in which he volatilized chloride of aluminium in the presence of sodium, operating in quantities of the chloride about half a pound in weight. By this means he obtained a double chloride of aluminium and sodium, which is fusible and capable of being converted into a vapor, if a sufficient heat be employed. He then distilled this at a red heat, bringing its vapor in contact with hydrogen gas, and obtained the aluminium in globules, which he reduced to a mass by fusing it in a porcelain crucible, with some of the double salt from which it was originally obtained.

The matter now attracted the attention of the emperor, who put at the command of the chemist more extensive appliances for experiment, in the manufactory of chemical products at Javel. Here, the operations were no longer confined to tubes and porcelain boats, but conducted on a comparatively large scale in furnaces prepared for the purpose. Alumina, obtained by heating ammonia-alum to redness, was mixed with coal tar and calcined. The mass was introduced into a gas-retort and subjected to the conjoint action of chloride and heat. The chloride of aluminium was converted into vapor as soon as formed, and condensed in a chamber of brick-work lined with delft. Thus obtained, it was in the form of a compact mass of sulphur-colored crystals, comparatively free from iron. It is further purified by passing the vapor over iron points, which reduced the volatile perchloride of iron to the more fixed protochloride of that metal. The chloride thus obtained, was subjected to the action of sodium in tubes.

At the time when these operations commenced, sodium was worth 1,000 francs the kilogramme, or nearly $100 a pound, making aluminium cost nearly $300 a pound. It became necessary, therefore, to cheapen the production of sodium. He effected this by means of an apparatus for continuous distillation, using a mixture of dry carbonate of soda, chalk, and well dried mineral coal. Dumas announced to the Acad my that the result of these experiments was to reduce the cost of the materials necessary for making alumina to 32 francs the kilogramme, or about $4 a pound. The sodium was obtained by this process, in blocks a cubic foot in diameter, as cheaply as it it could have been procured before in quantities of a few grains in weight.

In spite of this great improvement, however, the process for obtaining aluminium was still too intricate for the ordinary workmen. The chloride was introduced into a wide tube between plugs of asbestos and then heated in an atmosphere of hydrogen to drive off impurities. Porcelain boats containing sodium were then introduced, the tube heated till the sodium fused and the chloride of aluminium became vapor and was decomposed by the sodium. As soon as all the sodium had disappeared, the boats were withdrawn, the double chloride driven off by heat, and the remaining aluminium washed and refused with a quantity of the double chloride in a porcelain crucible. This process was slow, tedious, intricate and costly. By it the metal could not be sold for less than two francs the gramme, or $182 a pound. It is evident that a further economy was necessary before the new metal could come into general use.

The first reduction of cost was effected by discarding the ammonia alum and using kaolin or ordinary pure clay. Next the whole process of manufacturing the chloride of aluminium was set aside. This substance presented some difficulties in the manufacture, arising from its rapid condensation. It was necessary to collect in chambers and detach it mechanically from their sides, which involved according to Dumas, three inconveniences: 1st, loss of chloride, the condensation being incomplete; 2d, danger to the workmen exposed to its vapors; 3d, increase of expense on account of the disconnected character of the operations. Again, the production of sodium has been greatly cheapened. By the continuous process it can be made at a cost of about $3½ a pound, with the greatest facility; its manufacture, according to Dumas, presenting no more difficulty than that of zinc. The mixture of carbonate of soda, chalk and bituminous coal is so thoroughly decomposed, that it gives results corresponding to those of chemical calculation, and so easily managed that it can be worked in luted earthen tubes, instead of the expensive iron bottles formerly used.

The present process, as we have said, discards the chloride of aluminium. In the place of this, it substitutes the double chloride of aluminium and sodium, or sodio-chloride of aluminium. This is made by acting with chlorine and heat upon a mixture of alumina, common salt and charcoal. The double chloride passes over in the form of vapor, is condensed to a liquid, and subsequently hardens to a solid as it cools. Its preparation, like that of sodium, is continuous, going on with regularity, and requiring no further care than that necessary to produce the chlorine, and shift the earthen pots in which the chloride, flowing in a steady stream, is formed into cakes.

The double chloride is now broken up, mixed with fragments of sodium, and changed by the shovel full, into a heated reverberatory furnace by means of shovels. The reaction takes place slowly and without danger. The aluminium is obtained in plates, globules or powder, in either form, it is easily separated from the common salt, either mechanically or by means of water. The cost is still higher than it ought to be. Dumas thinks it could be made, under favorable circumstances, at 100 francs the kilogramme, or something less than ten dollars the pound. The little furnaces now at work turn out about four and half pounds a day.

As thus prepared, aluminium is a white metal, somewhat resembling silver in color, lustre and conducting power. Its specific gravity is variously stated at from 2.25 to 2.56. This can be increased by rolling to 2.64, and by hammering to 2.74. Its fusing point is intermediate between those of silver and zinc. It is powerfully electro-negative, rivalling platinum in this particular. Inserted in a galvanoplastic battery, by M. Hulot, a plate of this metal produced as strong a current with one of zinc, as platinum would have done. After six hours the current had lost a fifth of its original force. At the end of 24 hours, it still retained a fourth. To restore its electro-negative character, it was only necessary to immerse it an instant in nitric acid and then wash it. It is the most sonorous of all pure metals.

Chemically, it is an exception to the earthy metals. So far from greedily absorbing oxygen, it is acted upon with difficulty by that element. It does not tarnish in the atmosphere and retains its brilliancy even after having been exposed to the powerful oxydizing current of air which passes through a heated muffle. Sulphuric and nitric acids exert but a slight action upon it, but muriatic acid dissolves it readily, with the evolution of hydrogen gas. It has been suggested that aluminium on coming in contact with the atmosphere is immediately covered with a fine coating of oxyd, a thin film of transparent sapphire, which protects it against any farther action. This view is strengthened by the results which follow the combustion of a leaf of aluminium in the oxydizing flame of the blow-pipe. It appears to be converted into a mass of alumina, but when rubbed in an agate mortar, metallic particles appear, showing that the oxydation has been confined to the surface.

We are told that the pure aluminium is easily distinguished from the impure by its greater whiteness and its indistinct traces of crystallization. Generally, only one or two hexagons can be observed on the upper surfaces of pure ingots. The impure metal, on the contrary, has a bluish tint like zinc, and is covered on the surface with numerous circular crystals, radiating in different directions. It may be whitened by dipping it first in a concentrated solution of caustic alkali, and then into nitric acid. In working it, it is necessary to anneal it frequently.

Aluminium forms no amalgam with mercury, and refuses to form an alloy with either lead or antimony. Iron unites with it readily in all proportions, raising its fusing point, and forming in some proportions, crystalline compounds which of course destroy its malleability. It is freed from iron by fusing it with nitre. Zinc combines with it freely, lowering its point of fusion. Alloys of 100 of aluminium, with 10, 25, 150 of zinc, have been used as solders for the pure metal. Tin, even in small quantities, injures aluminium by rendering it brittle, while on the other hand, small quantities of aluminium give to tin the hardness and tenacity in which it is deficient. The alloys of tin, like those of zinc, have been used as solders, but are liable to the same objections, a fatty consistence and a fragibility which causes them to break under the hammer.

It forms many alloys with copper, small quantities of which impair its malleability, give it a bluish tinge, and a tendency to grow black in the air. In small quantities, aluminium increases the hardness of copper without greatly affecting its malleability, and produces alloys which very much resemble gold, and do not readily tarnish. The alloy of 100 copper with 10 of aluminium resembles exactly the pale gold of the jewelers, and may be hammered and worked like copper. If 100 of copper be mixed with 5 of the metal under consideration, an alloy is obtained more like pure gold. Silver increases the fusibility of aluminium, and in small quantities hardens it and increases its elasticity. Five per cent of aluminium hardens silver, and this alloy may be distinguished from one of copper with silver by the use of nitric acid, which turns the latter black and the former white. Equal parts of aluminium and silver form a brittle alloy.

Gold effects the ductility of aluminium less than any other metal. Small quantities of aluminium harden gold. The alloy of 5 parts aluminium to 100 gold, is white and as brittle as glass; with platinum it forms alloy, the fusibility of which varies directly with the proportion of aluminium.—*American Journal of Dental Science.*

